# Safety and efficacy of non-fluoroscopic endoscopic dilatation of gastrointestinal tuberculosis related strictures

**DOI:** 10.1186/s12876-022-02140-0

**Published:** 2022-02-11

**Authors:** Pankaj Kumar, Anuraag Jena, Chhagan Lal Birda, Harjeet Singh, Pankaj Gupta, Kaushal Kishor Prasad, Usha Dutta, Vishal Sharma

**Affiliations:** Departments of Gastroenterology, Gastrointestinal Surgery and Radiodiagnosis, Postgraduate Institute of Medical Education and Research (PGIMER), Chandigarh, India

**Keywords:** Intestinal tuberculosis, Abdominal tuberculosis, Surgery, Gastrointestinal tuberculosis, Colonoscopy, Crohn’s disease

## Abstract

**Introduction:**

Stricturing gastrointestinal tuberculosis (GITB) may result in persistent symptoms even after antitubercular therapy (ATT) and may require surgical intervention. Data on efficacy and safety of endoscopic dilatation for management GITB related strictures is scarce.

**Methods:**

A retrospective analysis of database of patients who underwent endoscopic balloon dilatation for suspected or proven gastrointestinal tuberculosis was performed. The analysis included the site of involvement, technical success, clinical success (response), relapse and requirement of surgery in these patients.

**Results:**

Out of 34 patients (47.1% males, mean age 31.9 ± 12.9 years), eventually four patients were diagnosed to have Crohn’s disease while the rest had GITB. Initial technical success was achieved in 30 (88.2%) patients. Initial clinical success was achieved in 28 (82.3%) patients. Median number of dilatation sessions required to obtain symptomatic relief were 2.5 (1–5) per patient. Two patients with initial clinical success had recurrence of symptoms over follow up of 1 year, out of which one patient was managed with repeat endoscopic balloon dilatation successfully. Of 30 patients with technical success, 16 (53.4%) were on ATT when they underwent dilatation while two were in intestinal obstruction. Eventually 7 patients required surgical intervention for various reasons.

**Conclusion:**

Non-fluoroscopic endoscopic balloon dilatation is an acceptable and fairly safe modality for symptomatic tuberculous strictures of gastrointestinal tract.

## Background

Tuberculosis (TB) of gastrointestinal tract (GIT) is an important form of extrapulmonary tuberculosis with diverse clinical presentations [1]. The luminal form of gastro-intestinal TB can have ulcerative, hypertrophic and stricturing variants and these could also occur in a combination. Strictures due to gastrointestinal tuberculosis (GITB) are typically described as short and concentric and may involve the colon, ileo-cecum, small intestine or the gastroduodenal area [1–3]. However, on occasion, mass forming disease may be confused with stricturing disease as the discrimination is not possible merely on endoscopic visualization. The clinical features of stricturing GITB depend on the site and severity of the lesion. While the patients with gastroduodenal tuberculosis mostly present with features of gastric outlet obstruction, tubercular strictures in the lower GI tract may be associated with abdominal pain and features of intestinal obstruction [3]. It is important to differentiate clinically relevant strictures (i.e. those causing symptoms) from those which may only be morphological without any functional impairment [4]. It has been noted in different studies that the clinical response and radiologic responses have different implications [5–8]. While the clinical symptoms may respond to antitubercular therapy (ATT), the radiological strictures may respond only partially [6, 8]. The clinical goal of endoscopic management of tubercular strictures is symptomatic relief and acceptance of normal diet. With the advent of endoscopic balloon dilatation (EBD), nowadays most of the patients with symptomatic strictures especially in inflammatory bowel disease are managed non-surgically [9]. Similar data is, however, scarcely reported for management of stricturing GITB. Surgery may be needed in cases where the stricture is either not endoscopically accessible or does not respond to dilatation.

There are certain issues regarding management of these strictures which are debatable and lack consensus. It is unclear which strictures are likely to be responsive to dilatation and what is the endpoint and final target size of dilatation. There are suggestions regarding the target final size of dilatation depending on the site of stricture (15 mm for esophagus and stomach, 18–20 mm for colon) but the evidence behind these suggestions is limited [10–12]. Also, the timing of starting dilatation especially in symptomatic patients who have been recently diagnosed is unclear. It is also unclear as to when should dilatation be done in patients on a diagnostic trial of ATT when a clear discrimination of intestinal TB and Crohn’s disease could not be made. In this retrospective study we analyze the feasibility and clinical success of endoscopic dilatation in patients who were clinically symptomatic because of diagnosed or suspected tuberculous gastrointestinal strictures.

## Methods

In this single center study, we retrospectively analyzed the data of patients who underwent endoscopic balloon dilatation (EBD) for symptomatic, proven or suspected tubercular strictures of GIT in between January 2016 to December 2020, at a large tertiary care institute. Those patients where complete medical records were found and were included for final analysis. Study was conducted after obtaining ethical clearance from Institute’s ethics committee and waiver of the requirement of informed consent was taken in view of retrospective nature of the study. All these patients were offered EBD as the first line treatment for their symptoms. Patients with age more than 12 years with symptomatic suspected or confirmed GITB related strictures who were either receiving or had completed antitubercular therapy (ATT) were included in the study. Patients with confirmed diagnosis of Crohn’s disease (except those initially treated as suspected GITB), suspected or proven malignancy and those with imminent need for surgery because of non-relenting obstruction were excluded from the study. Detailed history and clinical findings of the patients were recorded in the proforma. Endoscopic details like timing of EBD with respect to ATT duration, number of endoscopic dilatation sessions required, maximum dilatation required for symptomatic relief, adverse events of dilatation and need for surgery were recorded.

### Endoscopy and endoscopic balloon dilatation

**Fig. 1:**
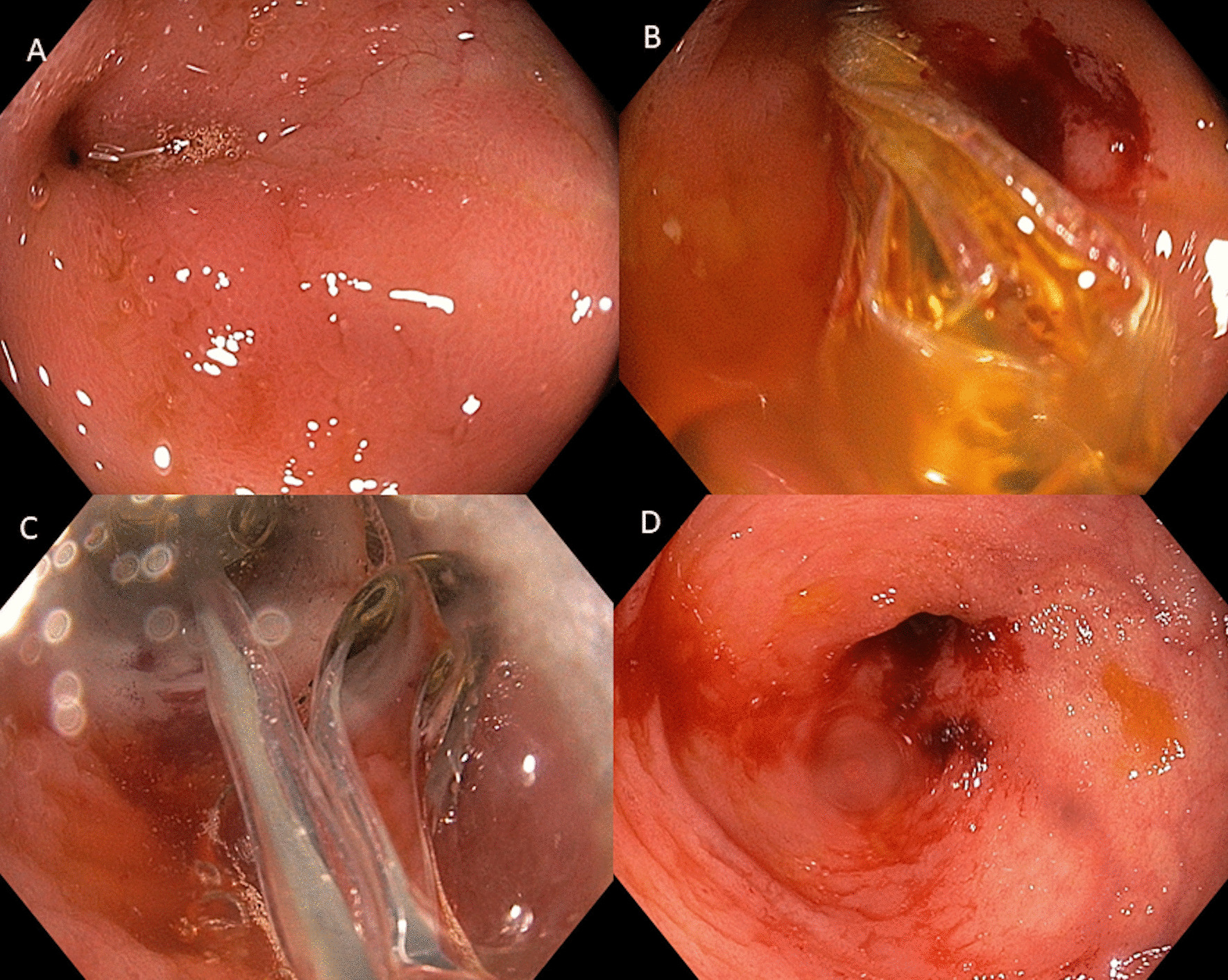
**A** Terminal ileal stricture. **B** Endoscopic through the scope balloon negotiated across the stricture. **C** Stricture dilatation. **D** Post dilatation

**Fig. 2:**
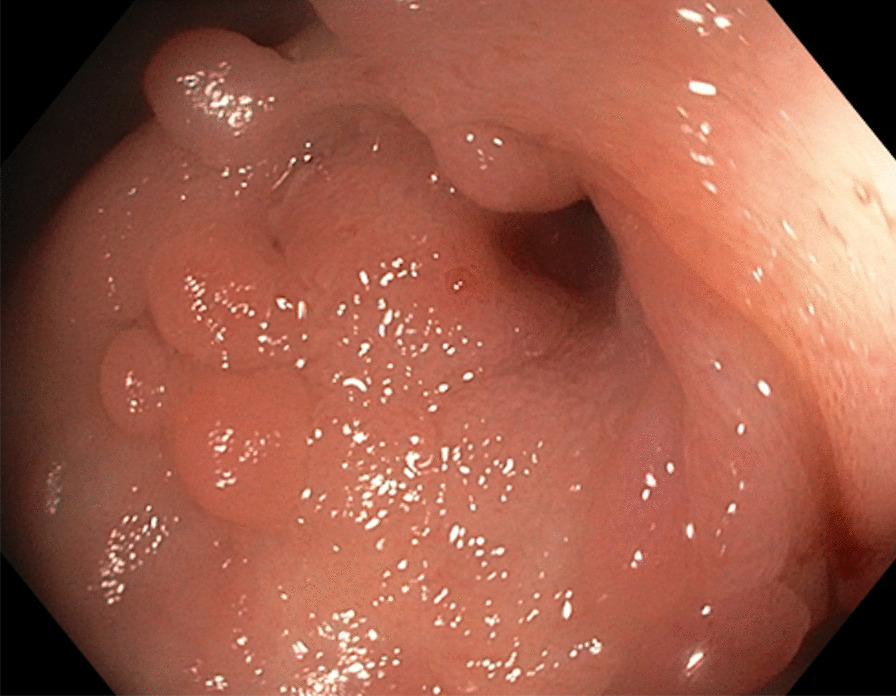
Ileocecal stricture which could not be negotiated with a balloon

In all enrolled patients in this study, the procedures were done after obtaining informed written preprocedural consent, endoscopy (Upper GI and Lower GI endoscopy) was carried out with an Olympus GIF 190 H endoscope with CV 190 processor, CLE 190 light source with high-definition video monitor after standard sterilization and rinse with sterile normal saline. Patients with strictures in the stomach or duodenum were taken for UGI endoscopy after fasting of 8 h. Patients for colonic dilatation were taken after standard split dose colonoscopy preparation using polyethylene glycol. All symptomatic patients who had evidence of stricture on imaging or on endoscopy when the endoscope could not be negotiated across narrowing were included. The endoscopist was aware of the clinical details of the patients. Endoscopic balloon dilatation was done using through the scope balloon (controlled radial expansion, TTS-CRE balloon, Boston Scientific, India) after premedication with intravenous midazolam, in symptomatic patients where we found any impassable stricture in the colon/terminal ileum or stomach/duodenum. All procedures were carried out under endoscopic guidance without use of fluoroscopy in patients without demonstrable ulcerations at the mouth of the stricture. The initial diameter of the CRE balloon was selected based on the severity of stricture on endoscopic assessment. Once the required size of dilatation was achieved the balloon was maintained in the same position for 30 s (Fig. 1). Next session of balloon dilatation was repeated after 2 weeks based on the final dilatation diameter of 15 mm or symptomatic relief of the patient. Total number of sessions required was noted. After dilatation patients were observed for 6 h. The dilatation was judged technically successful if the stricture could be negotiated by the deflated CRE balloon over the guidewire and the endoscopist could proceed with the initial dilation. The strictures where the guidewire was not negotiable were considered failures and referred for surgery (Fig. 2). EBD was considered clinically successful if obstructive symptoms of the patient got relieved after one or more sessions of dilatation irrespective of radiological/endoscopic resolution of the stricture and the size of last dilatation. The duration of follow-up was recorded and only those patients where at least three months follow-up after the last dilatation was available were included.

### Statistical analysis

All data and information were acquired retrospectively and entered in Microsoft Excel format for analysis. The descriptive data was presented as percentages for categorical variables and mean ± SD for interval scale variables.

## Results

### Patients

There were 36 patients who had received antitubercular therapy and undergone endoscopic dilatation. However, the records of two patients were incomplete and these were excluded from analysis. Of the 34 patients with complete details, 18 (52.9%) were females. The mean age was 31.9 ± 12.9 years (14–61 years). All patients were symptomatic with either pain abdomen, recurrent episodes of bowel obstruction or gastric outlet obstruction and were not responding to medical management. Of the 34 patients of TB strictures, 7 were confirmed cases based on microbiological positivity whereas the rest 27 were diagnosed on basis of response to therapy. The microbiological positivity was by culture positivity in 2 patients, PCR positivity in 3, AFB stain plus culture plus PCR in 1, and AFB stain plus culture in 1 patient. Four patients were eventually diagnosed to have Crohn’s disease (CD) and had received antitubercular therapy for suspected GITB. The location of stricture was in terminal ileum in 13 (38.2%) patients, ileocaecal junction in 12 (35.2%) patients, ascending colon in 3 (8.8%) patients, transverse colon in 1 (2.9%) patient, rectum in 2 (5.8%) patients and duodenal strictures were seen in 3 (8.8%) patients. During endoscopy, all of these patients had a non-negotiable stricture.

### Success and outcomes

**Fig. 3:**
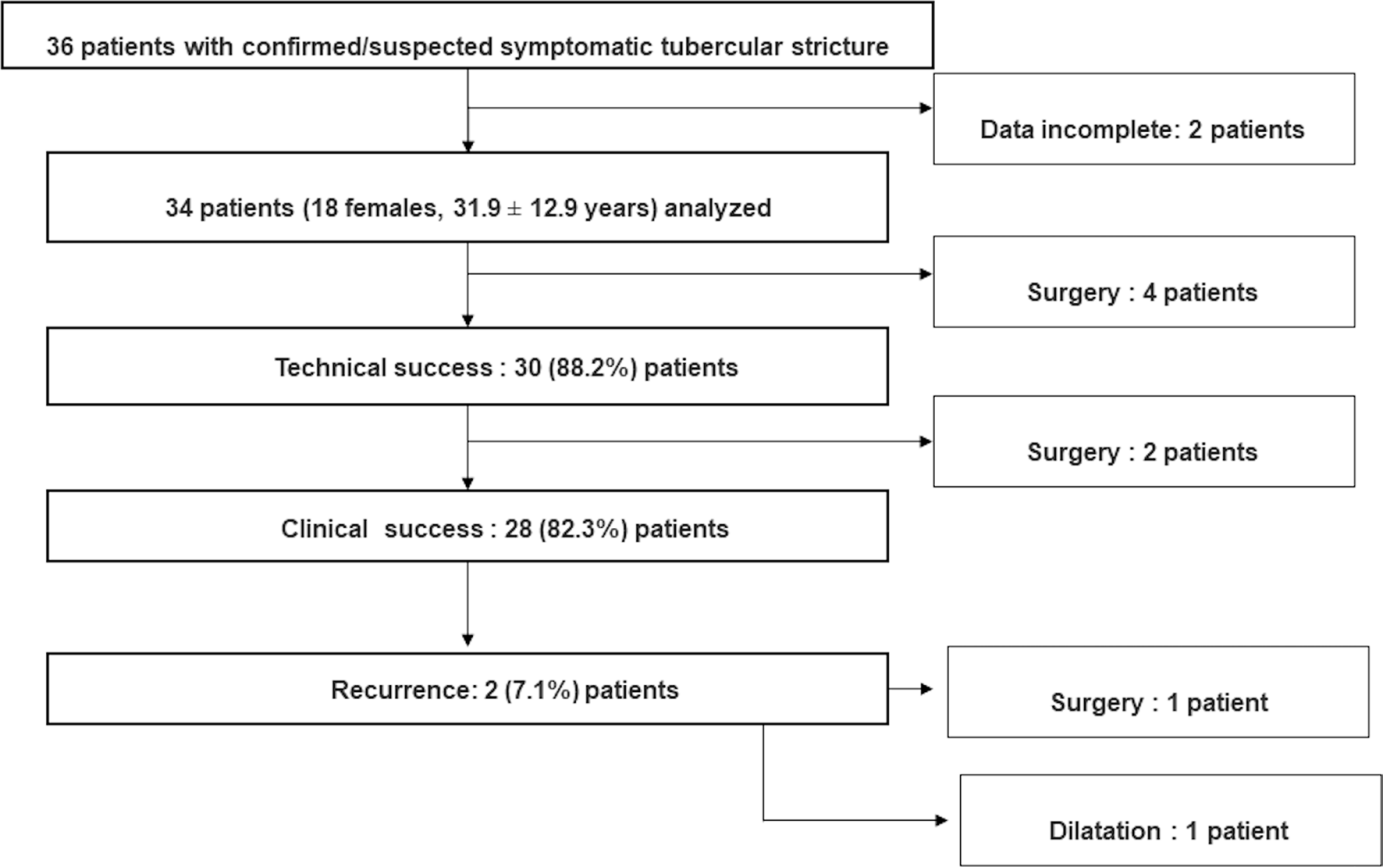
Flow chart showing the flow of the patients with clinical success and need for surgery

In 4 patients (three having terminal ileal stricture and one having stricture at the ileocecal junction), TTS-CRE balloon dilator could not be passed across the stricture and this was considered as technical failure (11.8%). These patients were subjected for surgical management. Of this one patient had underlying CD whereas the rest three had tuberculosis (Table 1). Two of these patients had mass forming disease or long strictures at surgery. Therefore, initial technical success was achieved in 30 (88.2%) of patients. However, in one of the patients who underwent a successful dilatation initially, the balloon could not be passed in the second session and since the patient was symptomatic, he underwent surgery. One patient presented with abdominal pain 2 weeks after last dilatation and was found to have perforation peritonitis due to a perforation just proximal to the site of stricture and needed surgery. Therefore 28 (82.4%) patients achieved initial clinical success. The median number of dilatation sessions required were 2.5 per patient to obtain symptomatic response (range, 1–5). These patients were followed every 2 weeks for resolution of clinical symptoms and were subjected for the next session of dilatation if symptoms persisted. Twenty-eight patients remained symptom free for 3 months after the last session of dilatation. Two patients out of 28 in which clinical success was achieved had recurrence of symptoms on long term follow up of 1 year. Out of this 1 patient was managed with repeat EBD, whereas the other underwent surgery as he was found to have CD related long stricture. Finally, 26 patients remained symptom free in the long term and 27 patients could be managed by endoscopic dilatation. Surgery was therefore needed in 7 patients (Fig. 3). Table 1 shows the outcomes of endoscopic dilatation in definite GITB, probable GITB and Crohn’s disease related strictures.

**Table 1:** Outcomes of endoscopic balloon dilatation in patients with probable tubercular, definite tubercular and Crohn’s disease related gastrointestinal strictures

Category	Number of patients	Technical success (%)	Clinical success (%)
Probable tubercular strictures	23	21 (93%)	20 (86.9%)
Definite tubercular strictures	7	6 (85.7%)	5 (71.4%)
Crohn’s disease related strictures	4	3 (75%)	3 (75%)
Total	34	30 (88.2%)	28 (82.4%)
*P* value		.629	.588

### Timing of dilatation and complications

Of the 30 patients who underwent dilatation, 14 had completed antitubercular therapy for at least 6 months. Of the 16 who were dilated while on ATT, the earliest dilatation was done at 2 months of ATT. Two patients were dilated while they presented with subacute intestinal obstruction while on ATT. These were admitted under surgical care and were taken for colonoscopy after resolution of the episode. Both were discharged are successful initial dilatation. However, one patient developed a perforation two weeks after the second session of dilatation and needed surgery.

## Discussion

Tubercular strictures, a well-recognized manifestation of GITB, are primarily managed with ATT along with endoscopic balloon dilatation or surgery, if required. In this study we report the efficacy of endoscopic dilatation of symptomatic tubercular strictures presenting as pain abdomen and intestinal obstruction. The most common site of tubercular strictures was terminal ileum followed by ileo-cecal junction. The technical success of endoscopic balloon dilatation was 88.2% with clinical success of 82.3%. All strictures were assessed endoscopically and dilated with balloon without the need of fluoroscopy. Stricture dilatation is not only feasible but also helps avoid surgery in the majority of cases.

The data regarding response of tubercular strictures to ATT alone is variable. While the classical paper by Anand et al. [5–8] reported that 91% of patients showed clinical response and 70% strictures showed endoscopic resolution, the results in the series published later are less impressive. Tubercular strictures have poor response to anti-tubercular therapy with only one fourth showing radiological stricture resolution with drugs alone. Symptoms due to tuberculous stricture are present in almost half of patients despite therapy [6]. In another series 45% patients with stricturing GITB continued to be symptomatic even after ATT [8]. Therefore, such patients who do not respond completely to ATT alone will require additional therapeutic intervention.

Endoscopic dilatation, if feasible, would be a preferable option because of the less invasive nature of the intervention. The role of endoscopic balloon dilatation was successfully shown in four patients of ileal tubercular stricture without any fluoroscopic guidance [13]. Endoscopic dilatation was done in 12 patients and 7 patients needed surgery in a study of 128 patients of tubercular stricture [6]. Similarly, colonic tubercular strictures were dilated successfully in 22 (of total 130 GITB) patients and it helped increase the diagnostic yield by 23% post dilatation [14]. Unlike strictures associated with Crohn's disease, benign etiologies like tuberculosis may need less sessions of dilatation for complete resolution [15]. This is because the ATT takes care of the underlying disease process resulting in the abatement of ongoing inflammation. Together with previous studies, our study suggests the endoscopic balloon dilatation could be a safe modality with fairly good clinical success. In our study only one patient had a complication of delayed perforation which was managed by surgery. In the previous study of 37 gastroduodenal strictures dilatation, two patients had complications of perforation. While one was managed conservatively, the other one needed surgical intervention [16]. The low recurrence rates and good clinical outcomes are the advantages of endoscopic balloon dilatation in tubercular strictures. Surgery is reserved for a subset of patients with tubercular strictures who are symptomatic despite endo-therapy and complications of dilatation like perforation and bleeding [8]. It is important to mention that fluoroscopy could be a useful tool in avoiding complication and early recognition of complications. Guidelines suggest that fluoroscopy should be used for complex or multiple strictures in patients with Crohn’s disease and these could be valid also for patients with GITB related strictures [10]. Another important concern is the dilatation in indeterminate situations where the diagnosis between GITB and CD is unclear and diagnostic trial of ATT has been resorted to. In such patients we would suggest that dilatation be undertaken only if there are no ulcers at the mouth of the stricture. It may also be preferable to ensure mucosal healing beyond the visual range by use of biomarkers especially fecal calprotectin [17].

There are limitations of our study. First, this is a single center retrospective series therefore many of the clinical details like pre and post endoscopy imaging could not be analyzed. Second, stricture characteristics were not evaluated in all patients and therefore many patients with mass forming lesions were included. Further we have used only standard colonoscopes (and not pediatric colonoscopes) and therefore the study cannot comment on additional benefit by use of scope with lesser caliber. We limited our dilatation to a maximum of 15 mm even in colon although the expert recommendations do suggest that 18–20 mm CRE may be used in Crohn’s disease related strictures [10]. Also, since we did not include the non-negotiable asymptomatic strictures in the study, we are unable to comment on the natural course of such strictures and the response to ATT. Most of the patients did not have microbiological positivity, but this is true for the spectrum of GITB where the yield of microbiological tests is low and response to therapy has to be utilized for the diagnosis [1, 18, 19]. However, there are many strengths of this study because the study describes the flow of patients as seen in clinical practice and therefore captures the possible reasons for failure of dilatation. As a result, we also have some patients who were thought to have gastrointestinal tuberculosis but eventually were diagnosed to have Crohn’s disease. The study also defines the feasibility of early dilatation in these patients. We performed dilatation while patients were still on ATT in large number of cases and also as early as 2 months in one case. We cannot preclude the possibility of stricture resolution with ongoing ATT because the natural course was interrupted by dilatation done for symptomatic stricture while the ATT was ongoing. The tubercular intestinal ulcers are recognized to heal completely in a majority of patients at two months and therefore dilatation could possibly be safe after two months of ATT [18].

## Conclusion

The present study reveals endoscopic balloon dilatation is a modality of choice for patients with tuberculous strictures along with anti-tubercular therapy. Dilatation is usually safe and has an acceptable technical and clinical success on follow up.

## Data Availability

The data associated with this paper can be obtained from corresponding at reasonable request.
